# Supplementary material for: Deciphering DEL pocket patterns through contrastive learning
Source: Nat Commun. 2026 Feb 16;17:2810. doi: 10.1038/s41467-026-69663-y (PMC13022000; doi:10.1038/s41467-026-69663-y)
Supplement: Supplementary file 1 — Supplementary Information [file 41467_2026_69663_MOESM1_ESM.pdf]

Supplementary Information for
Deciphering DEL Pocket Patterns Through Contrastive Learning

Wenyi Zhang[1,2], Yuxing Wang[1,2], Rui Zhan[1,2], Runtong Qian[1,2], Qi Hu[1,2], Jing Huang[1,2,*].

1. State Key Laboratory of Gene Expression, School of Life Sciences, Westlake University, 600 Dunyu Road, Hangzhou, Zhejiang 310030, China.

2. Westlake AI Therapeutics Lab, Westlake Laboratory of Life Sciences and Biomedicine, 18 Shilongshan Road, Hangzhou, Zhejiang 310024, China.

*Corresponding author here

Email: huangjing@westlake.edu.cn

This PDF file includes:

Figures S1 to S19
Tables S1 to S2

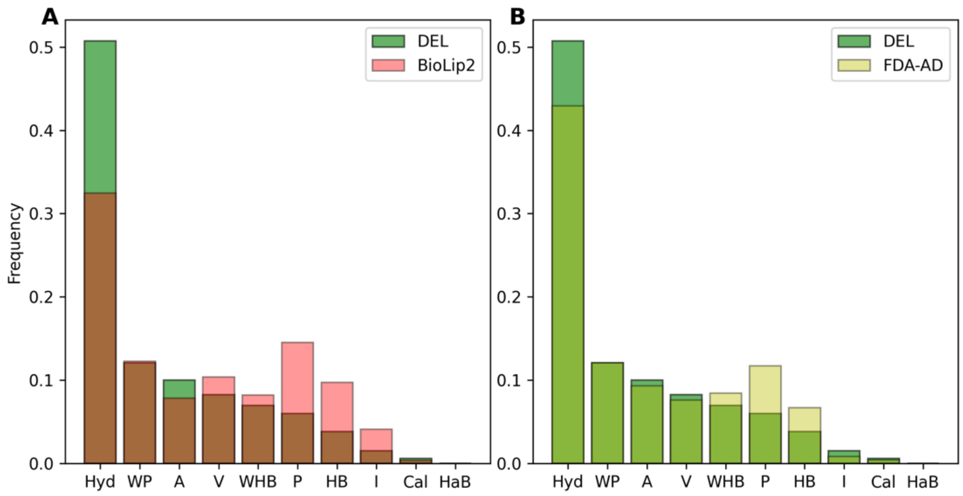

Figure S1. Comparison of 10 different types of interactions between pockets and ligands in the DEL, BioLiP2, and FDA-AD datasets. The interactions are represented by the following types: 'V' (Van der Waals), 'HB' (Hydrogen Bond), 'WHB' (Weak Hydrogen Bond), 'HaB' (Halogen Bond), 'I' (Ionic), 'A' (Aromatic), 'Hyd' (Hydrophobic), 'Cal' (Carbonyl), 'P' (Polar), and 'WP' (Weak Polar). The sample size (n) used for each comparison is reported in the corresponding Source Data file.

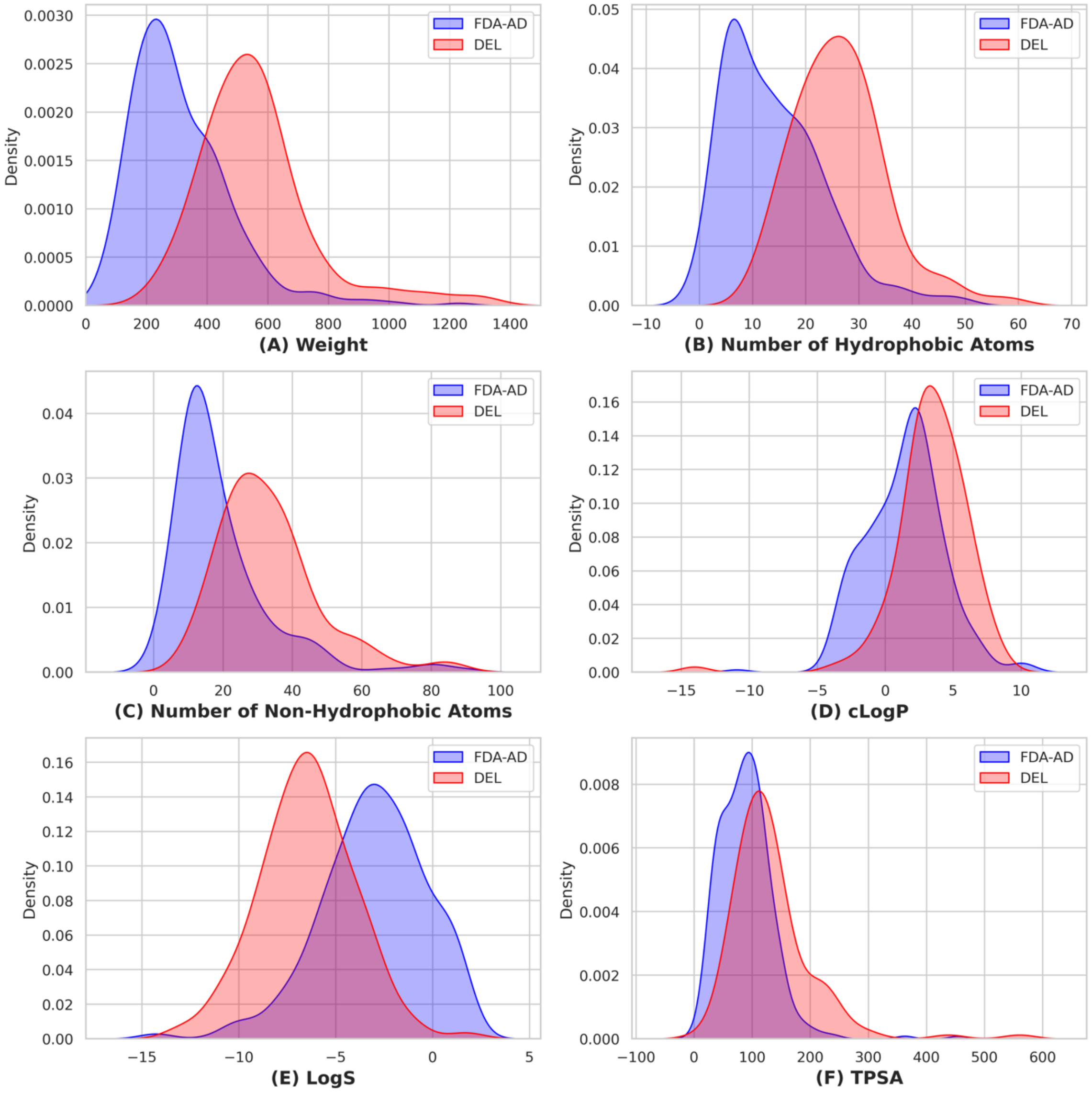

Figure S2. Comparison of physicochemical properties between 128 DEL molecules and 340 FDA-approved drugs. The properties analyzed include (A) molecular weight, (B) the number of hydrophobic atoms, (C) the number of hydrogen atoms, (D) the log octanol/water partition coefficient (cLogP), (E) the log solubility in water (logS), and (F) the topological polar surface area (TPSA).

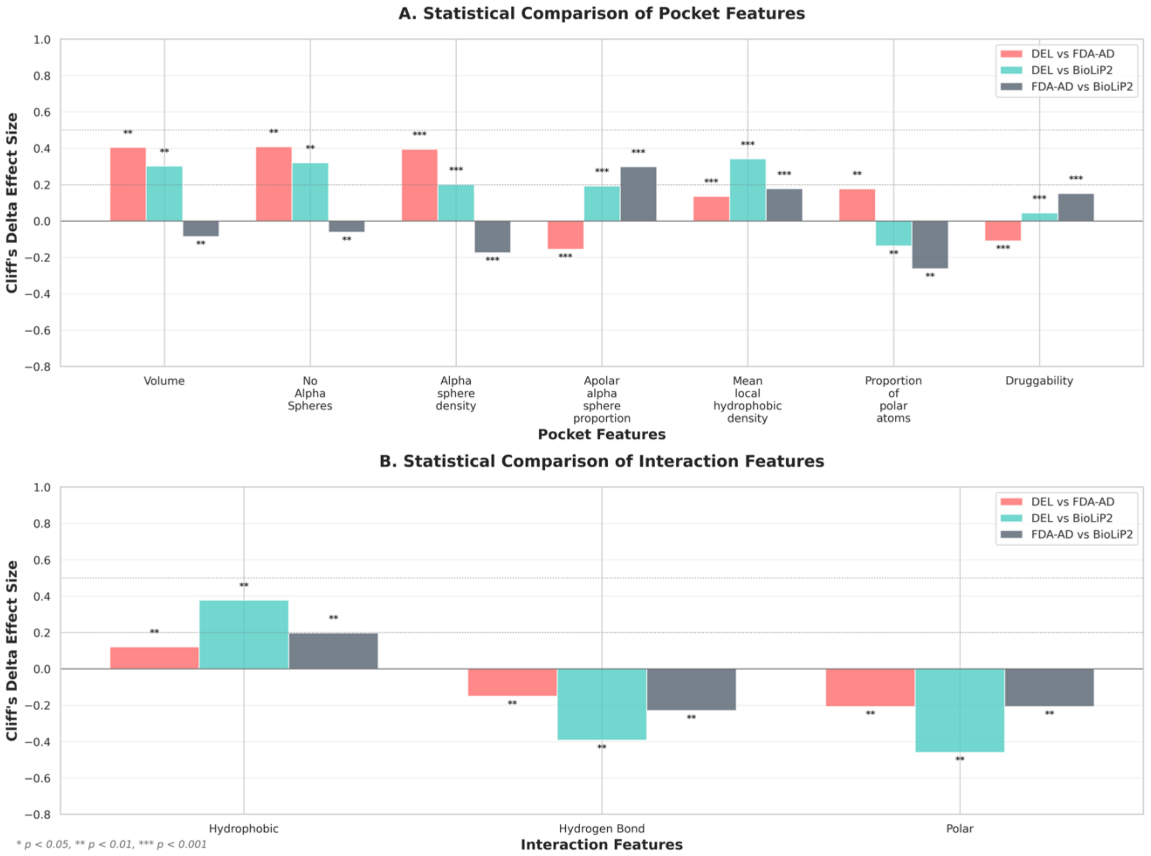

Figure S3. Statistical comparison of pocket and interaction features. A. Pocket features (volume, alpha spheres, density, polar/apolar composition, hydrophobicity, druggability) compared between DEL, FDA-AD, and BioLiP2 targets using Cliff's delta; statistical significance was assessed using two-sided Mann–Whitney U tests (* $p < 0.05$, ** $p < 0.01$, *** $p < 0.001$; uncorrected for multiple comparisons); **B.** Interaction features (hydrophobic, hydrogen-bond, and polar contacts) compared across the three datasets using Cliff's delta with the same significance notation. The sample size (n) used for each comparison is reported in the corresponding Source Data file.

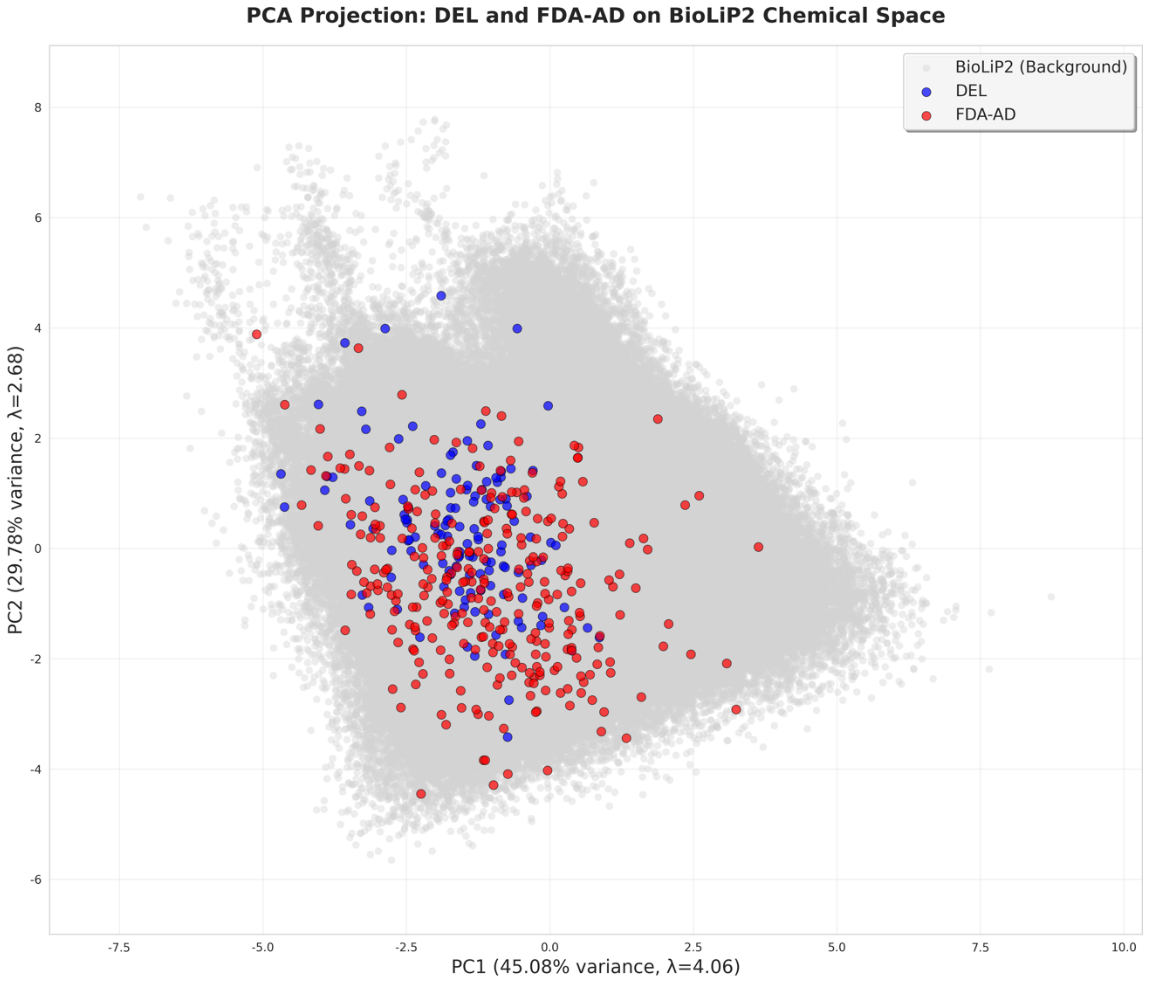

Figure S4. Principal component analysis (PCA) of pocket and interaction features. 128 DEL (blue) and 340 FDA-AD (red) targets were projected onto the PCA space trained using BioLiP2 pockets (gray background). Kernel density contours highlight the distinct yet partially overlapping distributions of DEL and FDA-AD targets. PC1 is primarily influenced by chemical composition, including apolar–polar balance and interaction types (e.g., apolar α-sphere proportion 4.1%, proportion of polar atoms 4.0%, hydrophobic 3.8%, hydrogen bonds 3.9%, polar interactions 4.0%), whereas PC2 is dominated by structural size descriptors (pocket volume 11.7% and number of alpha spheres 11.2%). Together, these components capture the separation of DEL targets from reference datasets.

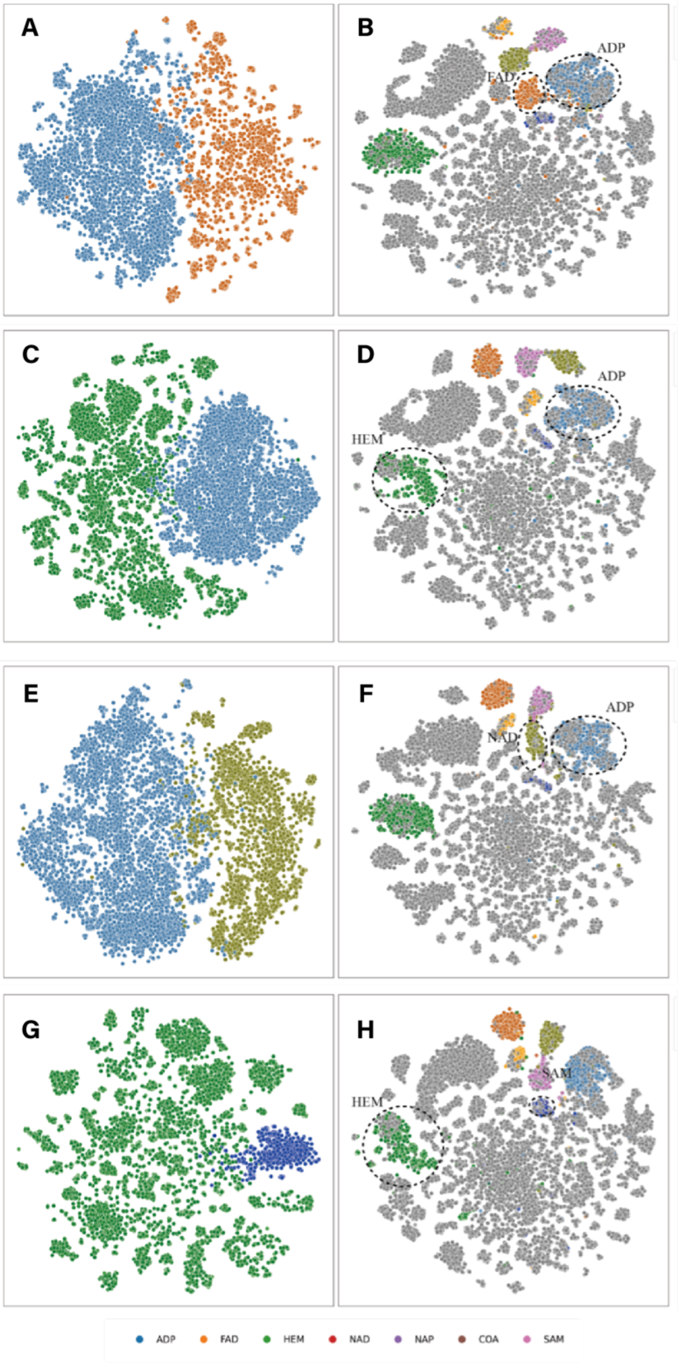

Figure S5. **t-SNE visualization of the model's performance in classifying excluded binding pocket types during the ablation study.** The study involved excluding different combinations of binding pockets from the BioLiP2 training dataset: ADP and NAD pockets (A and B), HEM and ADP pockets (C and D), ADP and NAD pockets (E and F), and HEM and SAM pockets (G and H). The excluded pocket types include ADP (n = 9,513), NAD (n = 5,354), HEM (n = 13,312), and SAM (n = 1,228).

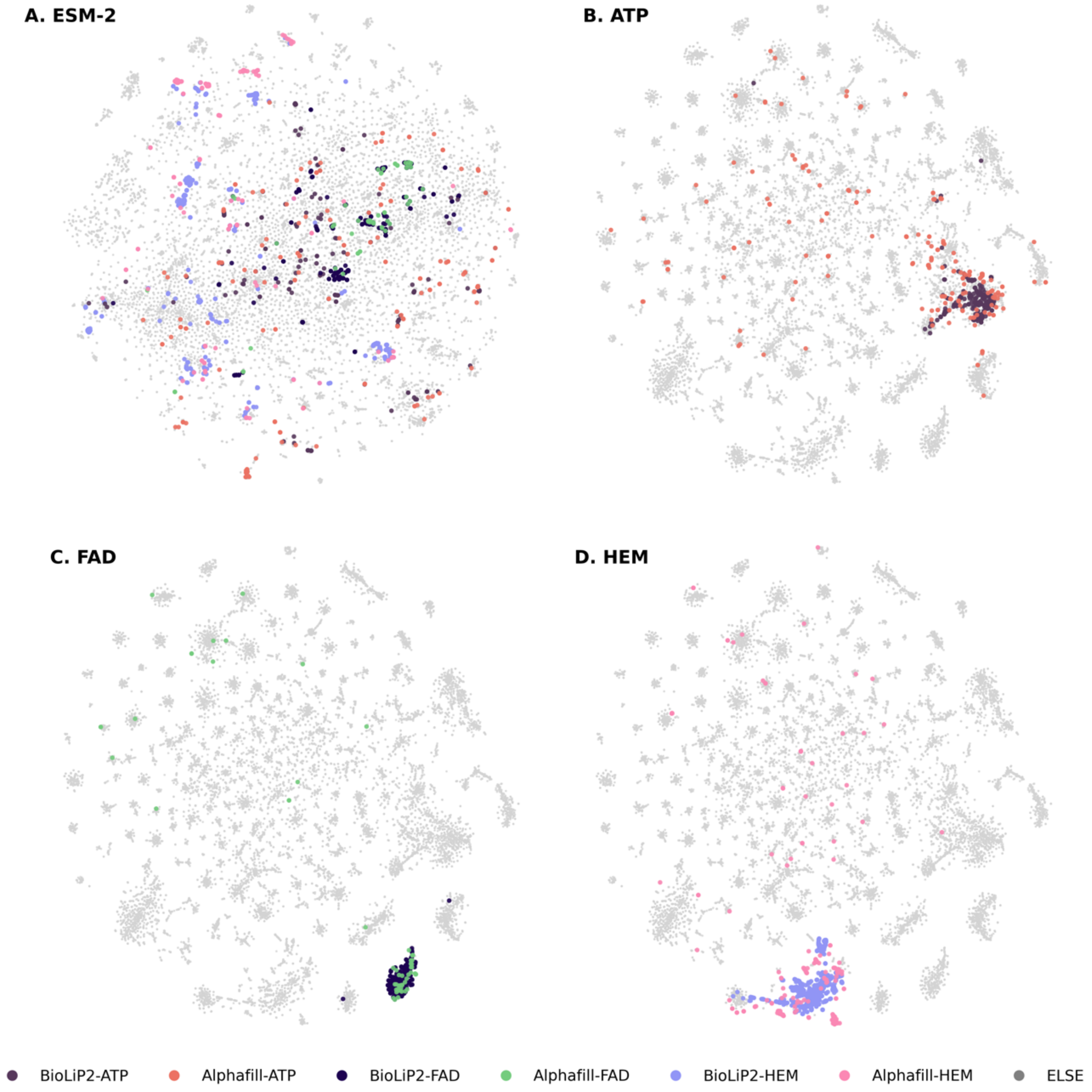

Figure S6. t-SNE clustering of ATP, FAD, and HEM binding pockets from BioLiP2 and Alphafill datasets. (**A**) Clustering with ESM2 features. (**B**) ErePOC clustering for ATP pockets. (**C**) ErePOC clustering for FAD pockets. (**D**) ErePOC clustering for HEM pockets. A total of 10,000 pockets were randomly selected from each of the BioLiP2 and AlphaFill datasets. Within these subsets, BioLiP2 pockets include ATP (n = 201), FAD (n = 358), and HEM (n = 718); AlphaFill pockets include ATP (n = 222), FAD (n = 58), and HEM (n = 226).

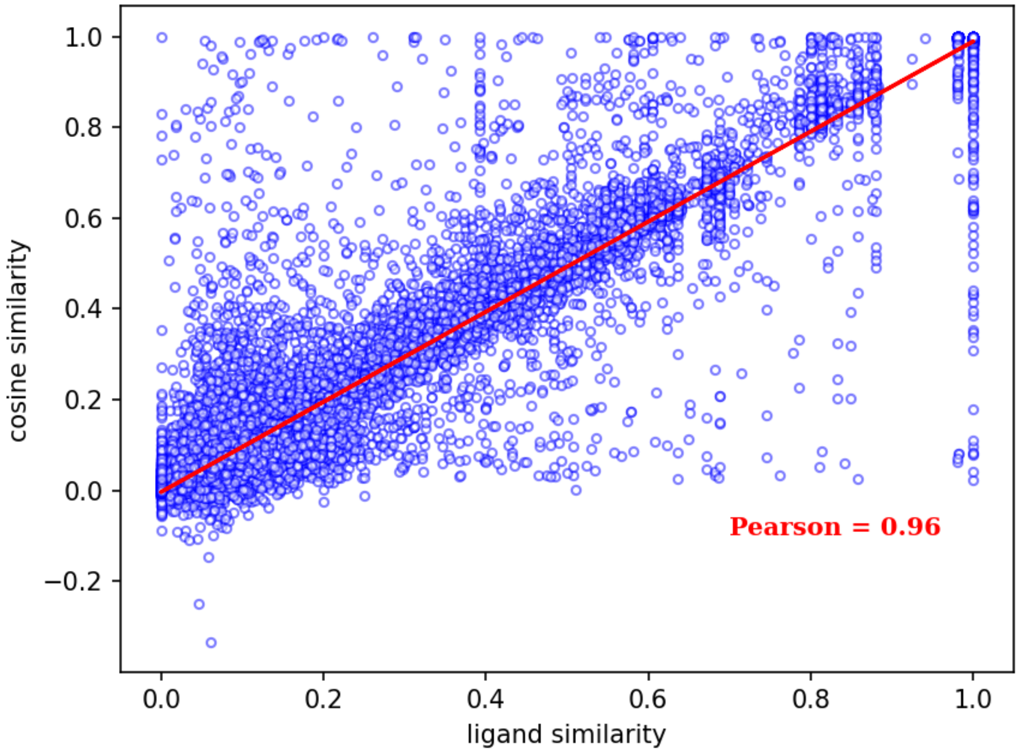

Figure S7. Pearson Correlation Analysis between ligand Tanimoto similarity and pocket cosine similarity derived from the ErePOC vector, based on 500 randomly selected ligand–pocket pairs. The Pearson correlation coefficient (r) is shown to quantify the strength of association.

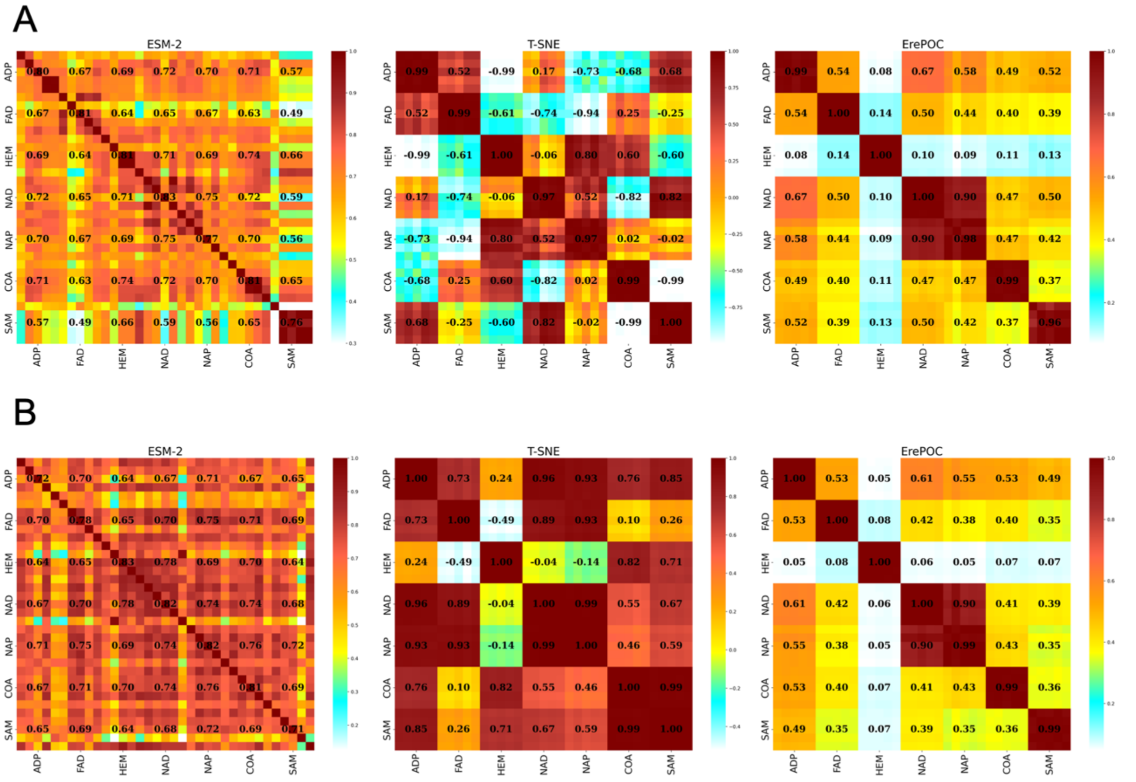

Figure S8. Cosine similarity comparison across different binding pockets under two BioLiP2 pocket definitions. A. Cosine similarity results based on the original BioLiP2 annotation–defined pockets, including ESM-2 vectors, t-SNE 2D representations after ErePOC transformation, and ErePOC embeddings, shown for illustrative examples based on five randomly selected pockets from each of the seven ligand-binding types; **B.** Corresponding results obtained using the unified 5 Å pocket definition. The overall similarity distributions remain consistent across the two definitions, confirming the robustness of ErePOC embeddings with respect to pocket boundary choice.

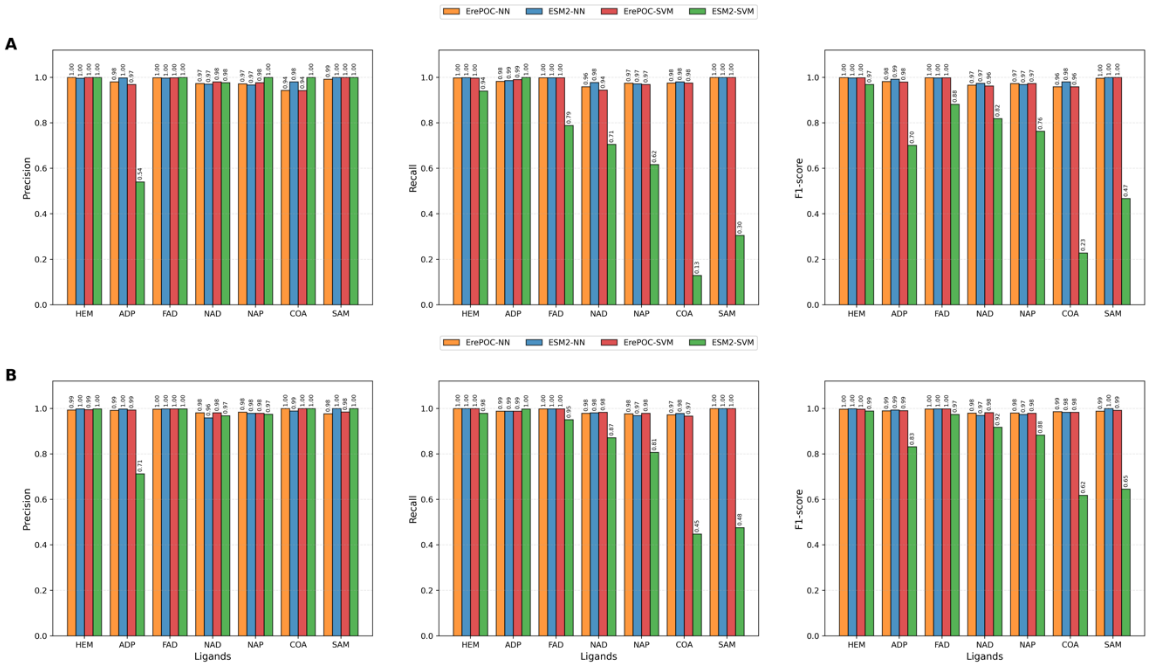

Figure S9. Comparative performance of four models under two BioLiP2 pocket definitions. A. Predictive performance of ErePOC-NN, ErePOC-SVM, ESM2-NN, and ESM2-SVM using BioLiP2 annotation–defined pockets; **B.** Corresponding results using the unified 5 Å pocket definition. The pocket types include ADP (n = 9,513), FAD (n = 6,367), HEM (n = 13,312), NAD (n = 5,354), NAP (n = 3,997), COA (n = 1,900), and SAM (n = 1,228).

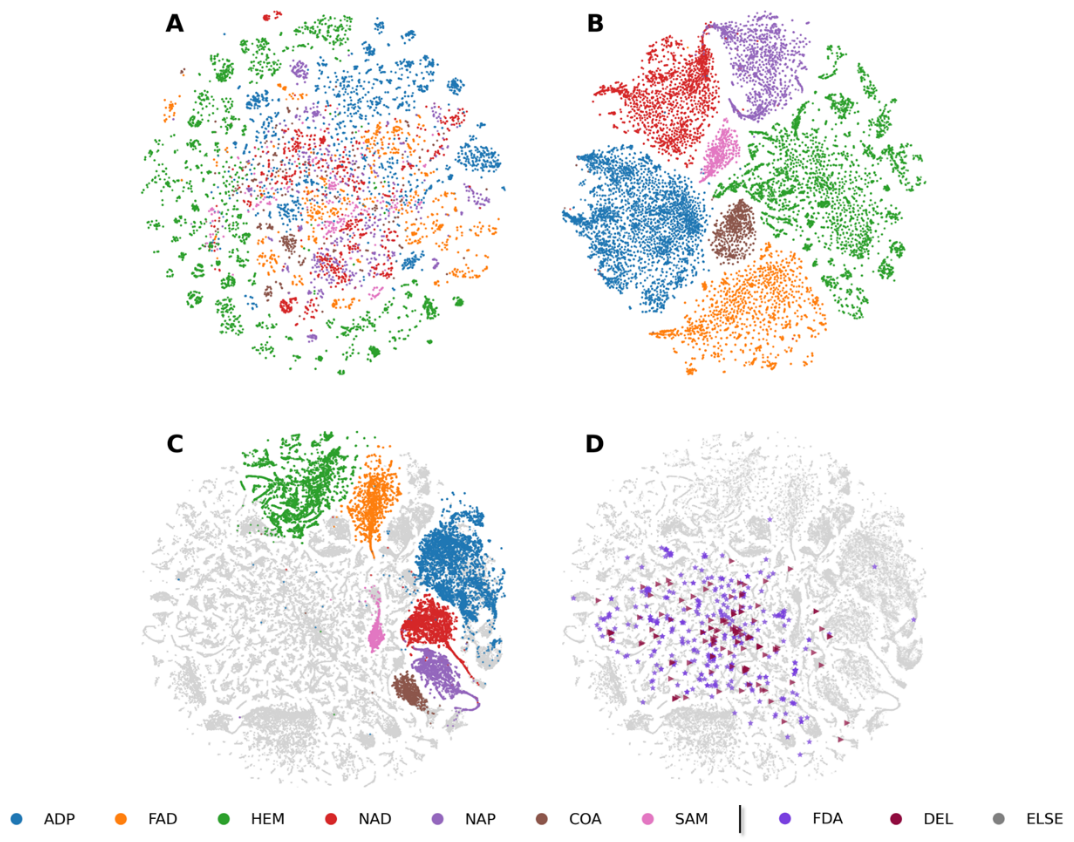

Figure S10. t-SNE visualization of ErePOC and ESM-2 representations for BioLiP2 dataset using the 5 Å pocket definition. A–B. Visualization of the seven types of ligand-binding pocket landscapes using ESM-2 (A) and ErePOC (B) representations, respectively. **C.** Pocket landscape using ErePOC representations. **D.** Comparison of pocket landscapes for the FDA-AD and DEL datasets against BioLiP2 using ErePOC representations. Results are consistent with those obtained using curated BioLiP2 binding annotations (Figure 4), confirming the robustness of the ErePOC embedding with respect to pocket definition. The pockets include ADP (n = 9,513), FAD (n = 6,367), HEM (n = 13,312), NAD (n = 5,354), NAP (n = 3,997), COA (n = 1,900), and SAM (n = 1,228); FDA-AD (n = 340) and DEL (n = 128).

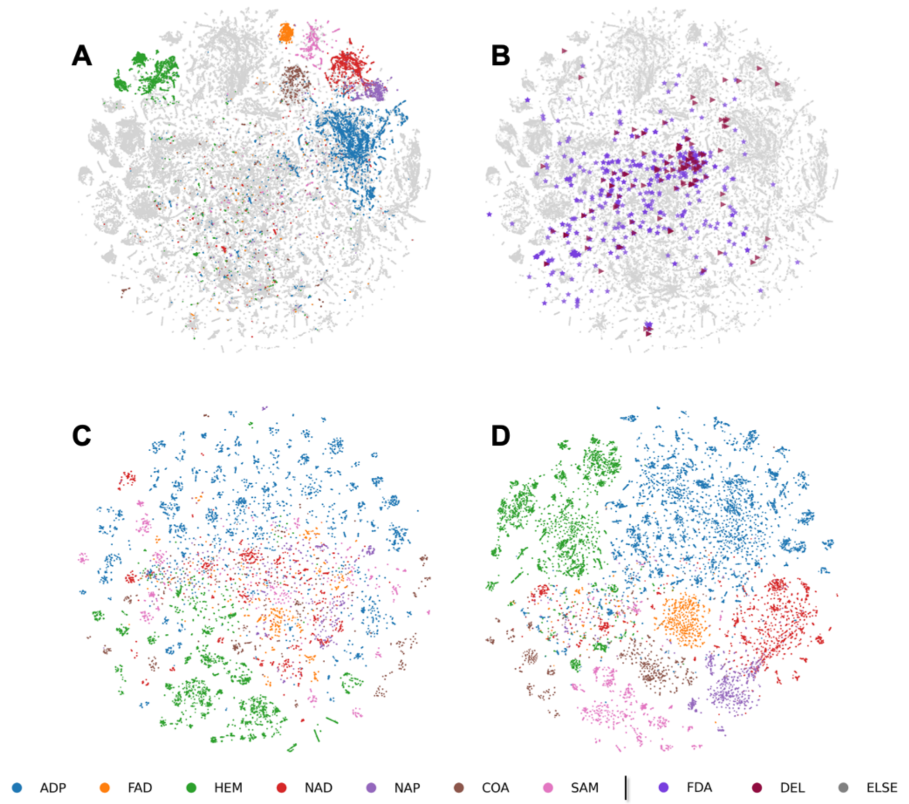

Figure S11. t-SNE visualization of ErePOC and ESM2 representations for AlphaFill dataset, where ErePOC was trained on BioLiP2 annotation–defined pockets. **A.** Pocket landscape using ErePOC representations. **B.** Comparison of pocket landscapes for the FDA-AD and DEL datasets against AlphaFill using ErePOC representations. **C-D.** Visualization of the 7 types of ligand-binding pocket landscapes using ESM2 (C) and ErePOC (D) representations, respectively. The pockets include ADP (n = 13,009), FAD (n = 1,852), HEM (n = 6,907), NAD (n = 4,409), NAP (n = 2,066), COA (n = 2,864), and SAM (n = 2,736); FDA-AD (n = 340) and DEL (n = 128).

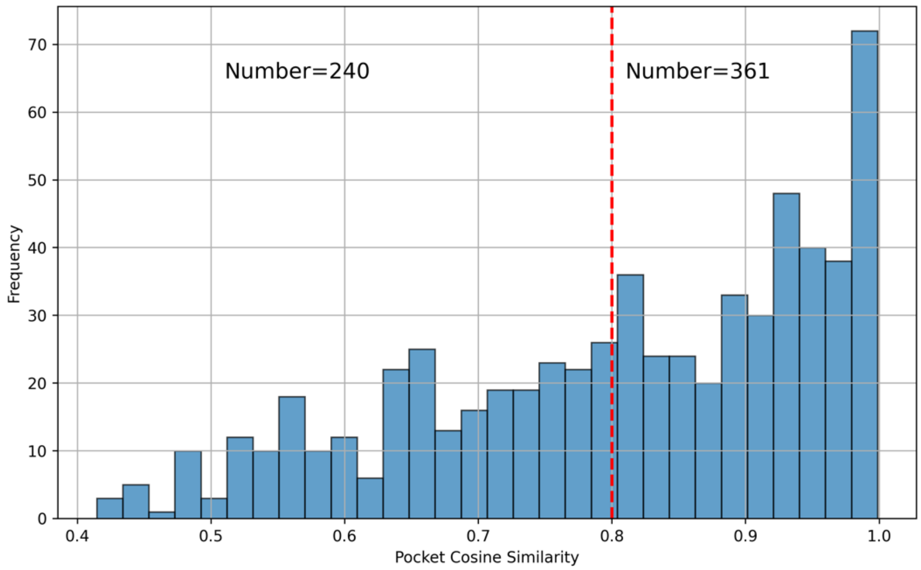

Figure S12. Distribution of DEL neighbor (n=601) cosine similarity scores in BioLiP2.

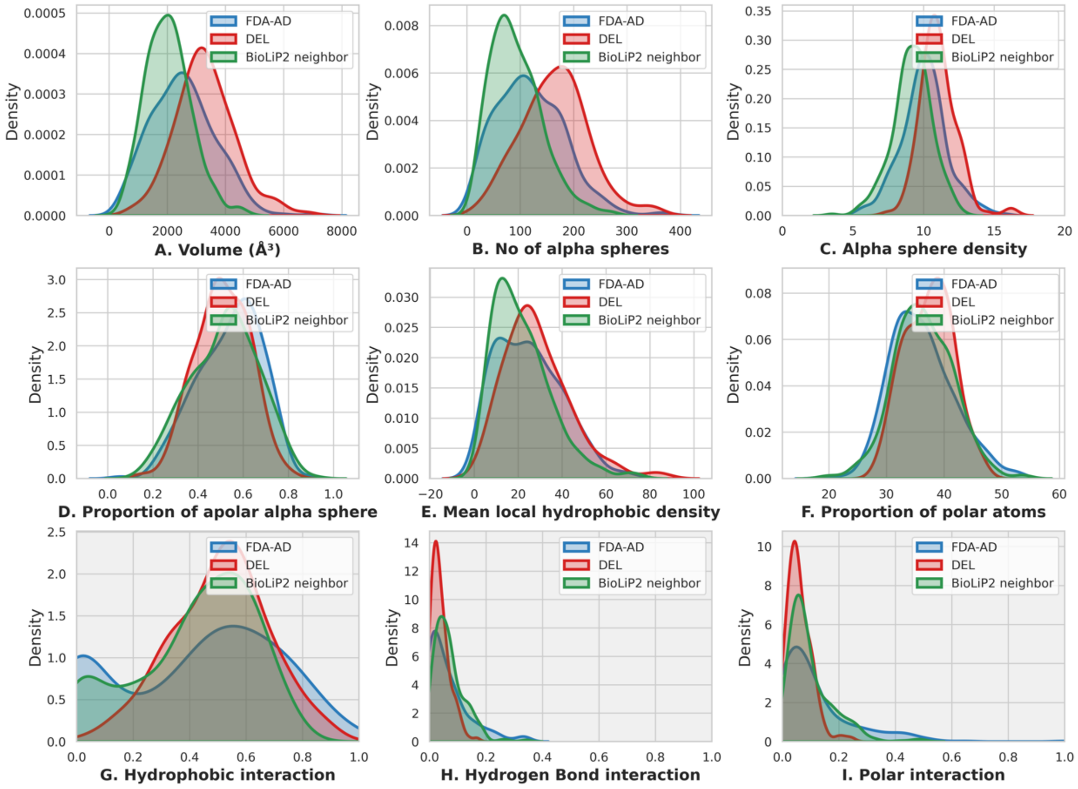

Figure S13. Physicochemical properties of pockets and ligand-pocket interaction analysis for DEL neighbors, comparing with DEL and FDA-AD targets. DEL neighbors are defined as BioLiP2 pockets with a cosine similarity greater than 0.8 to DEL pockets. **(A–F)** Panels show pocket physicochemical properties calculated using Fpocket, including volume, number of alpha spheres, alpha-sphere density, proportion of apolar alpha spheres, mean local hydrophobic density, and proportion of polar atoms. **(G–I)** Panels depict ligand–pocket interaction characteristics, including hydrophobic interactions, hydrogen bonds, and polar interactions. The sample size (n) used for each comparison is reported in the corresponding Source Data file.

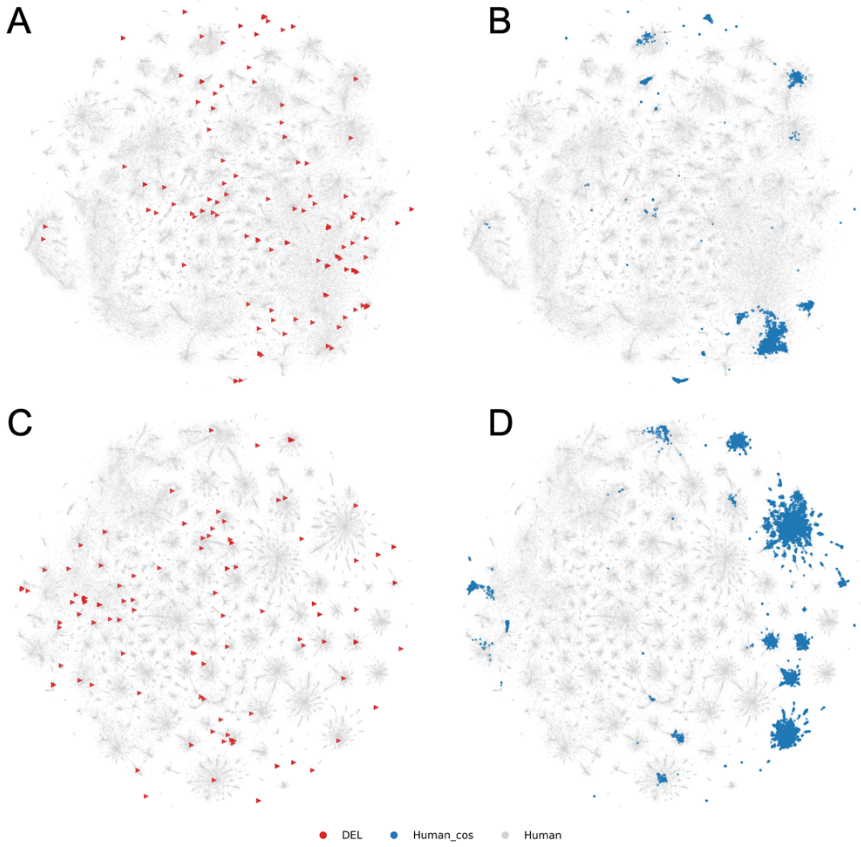

Figure S14. t-SNE visualization of DEL pockets and human pockets with cosine similarity > 0.8. A-B. Results obtained using the model trained on BioLiP2 annotation–defined pockets. **C-D.** Results obtained using the model trained with pockets defined as all residues within 5 Å of the bound ligand. The DEL dataset contains 128 pockets; panels B and D include 4,774 and 10,956 human pockets with cosine similarity > 0.8, respectively.

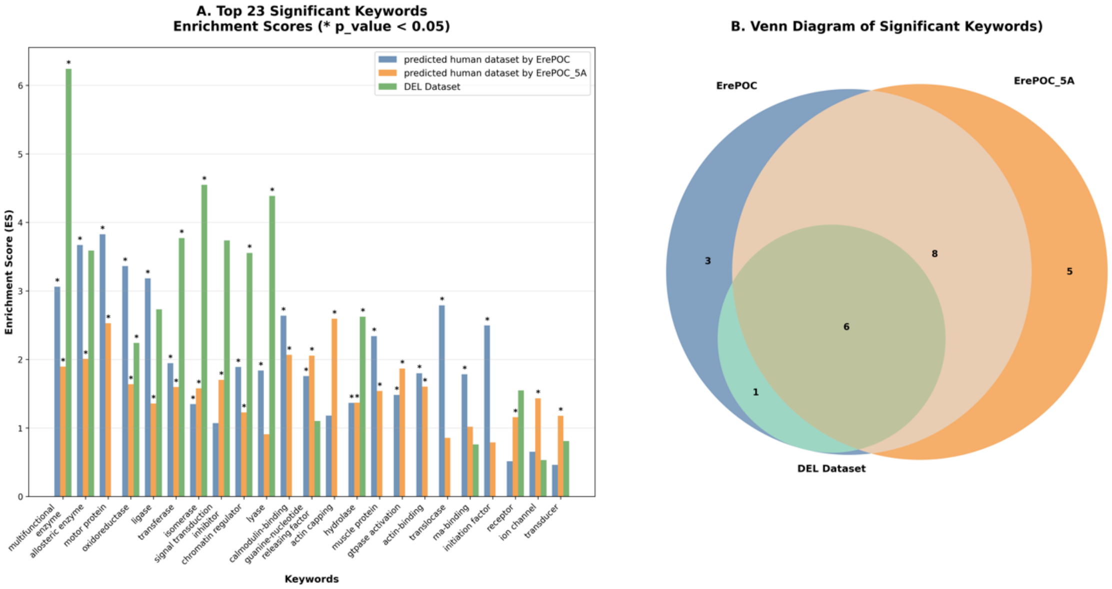

Figure S15. Comparative analysis of significantly enriched keywords across predicted human proteome datasets and DEL dataset. A. Top 23 significantly enriched keywords showing enrichment scores (ES) for predicted human dataset by ErePOC (blue), predicted human dataset by ErePOC_5A (orange), and DEL dataset (green). Asterisks (*) indicate statistical significance (p-value < 0.05), assessed using a one-sided hypergeometric test for over-representation relative to the corresponding background protein sets. The ErePOC and ErePOC_5A models yielded 2,739 and 5,405 unique predicted human proteins, respectively. **B.** Venn diagram illustrating the overlap of significantly enriched keywords among the three datasets, demonstrating the shared and unique functional features identified by each prediction method. ErePOC_5A means the model was trained using the 5 Å pocket definition.

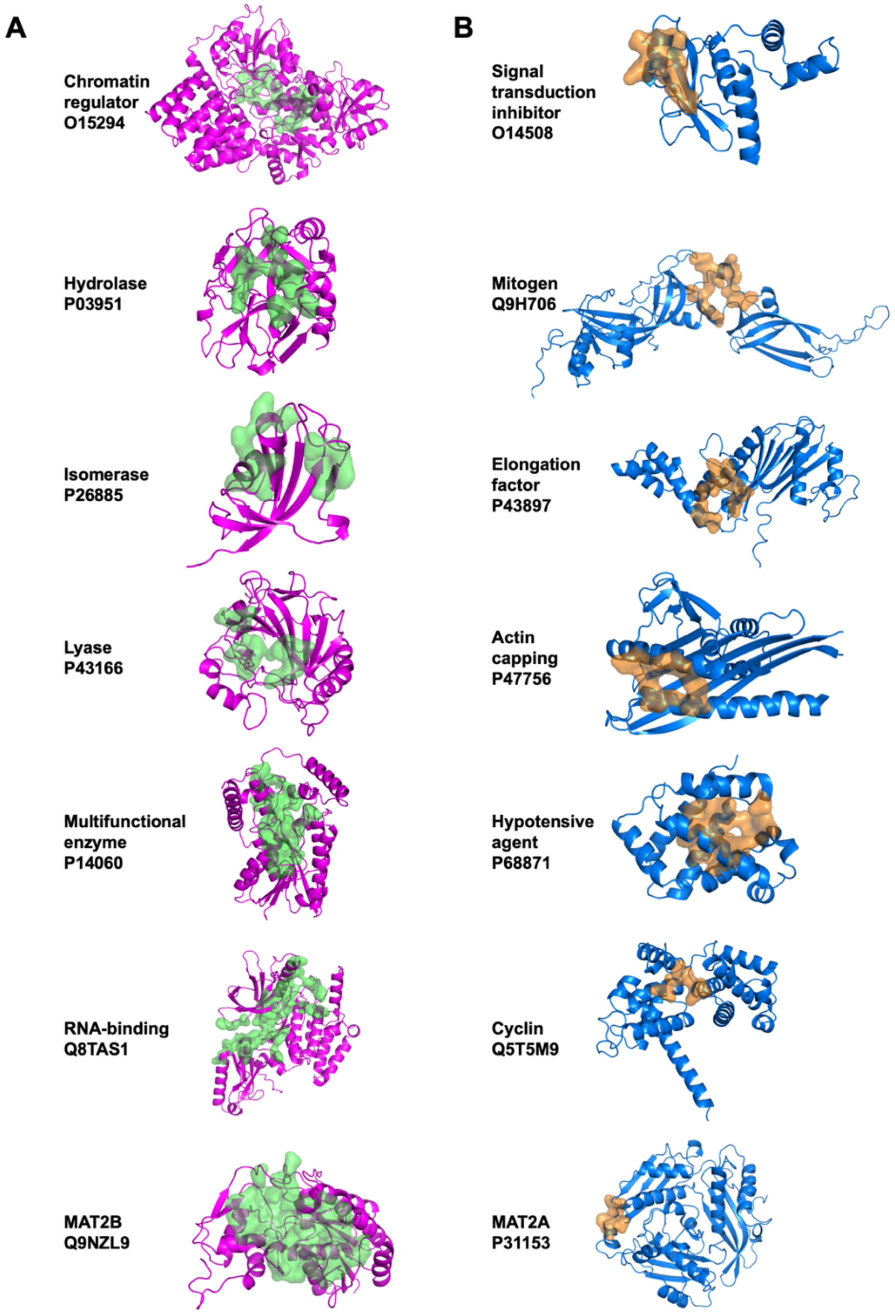

Figure S16. Selected human proteins and their pockets for in silico DEL screening. This figure shows the 14 human protein targets selected for large-scale virtual screening on a DEL compound library, grouped into DEL-enriched (left panels, A) and DEL-neutral (right panels, B) categories based on computational predictions. The MAT2B and MAT2A (bottom panels) are included as an example for different predicted DEL compatibility within a single protein family. These classifications support the experimental design used to evaluate ErePOC-predicted DEL suitability.

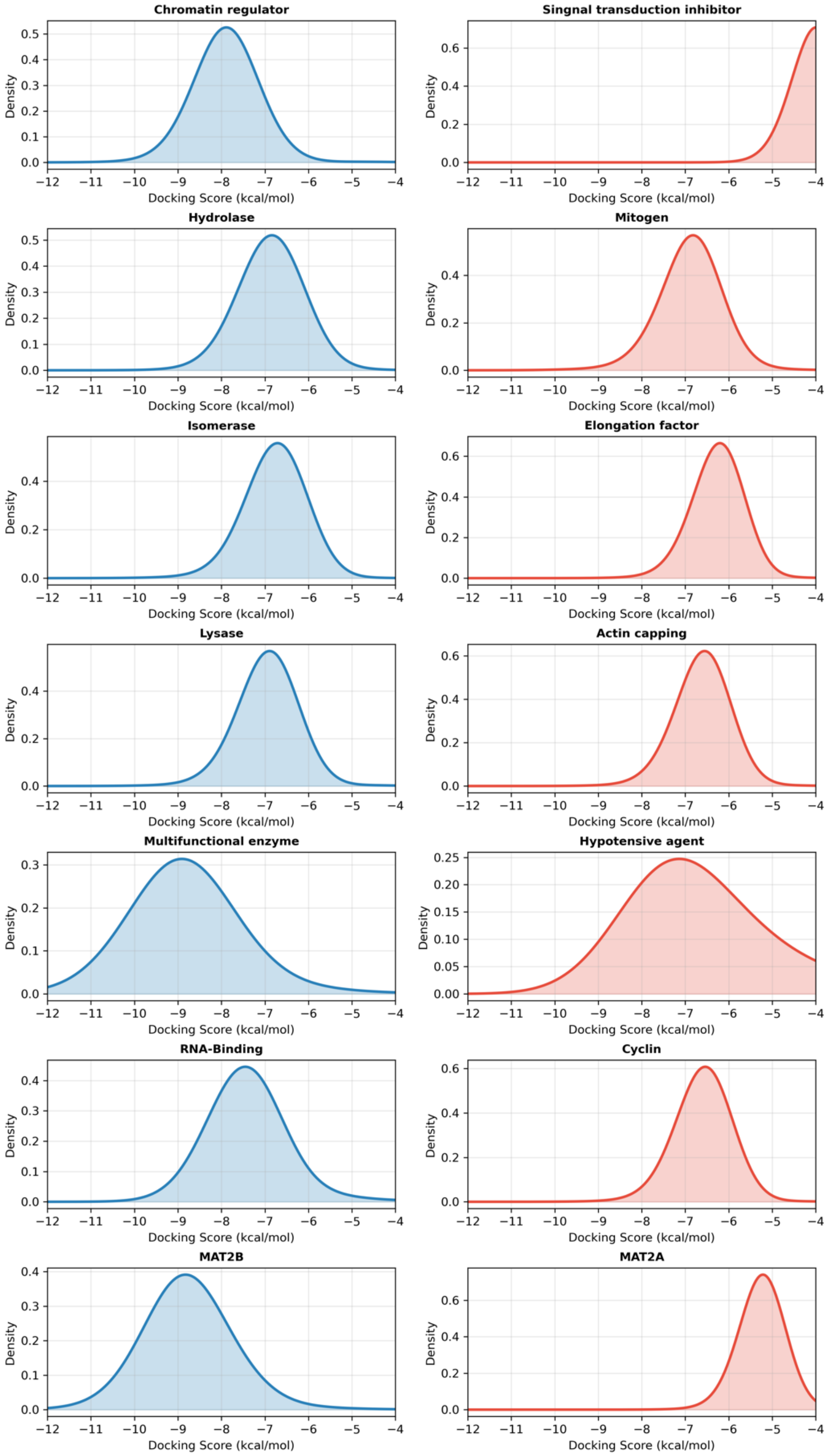

Figure S17. Docking score distributions for the 14 human protein targets against a library of 2.8 million small molecules.

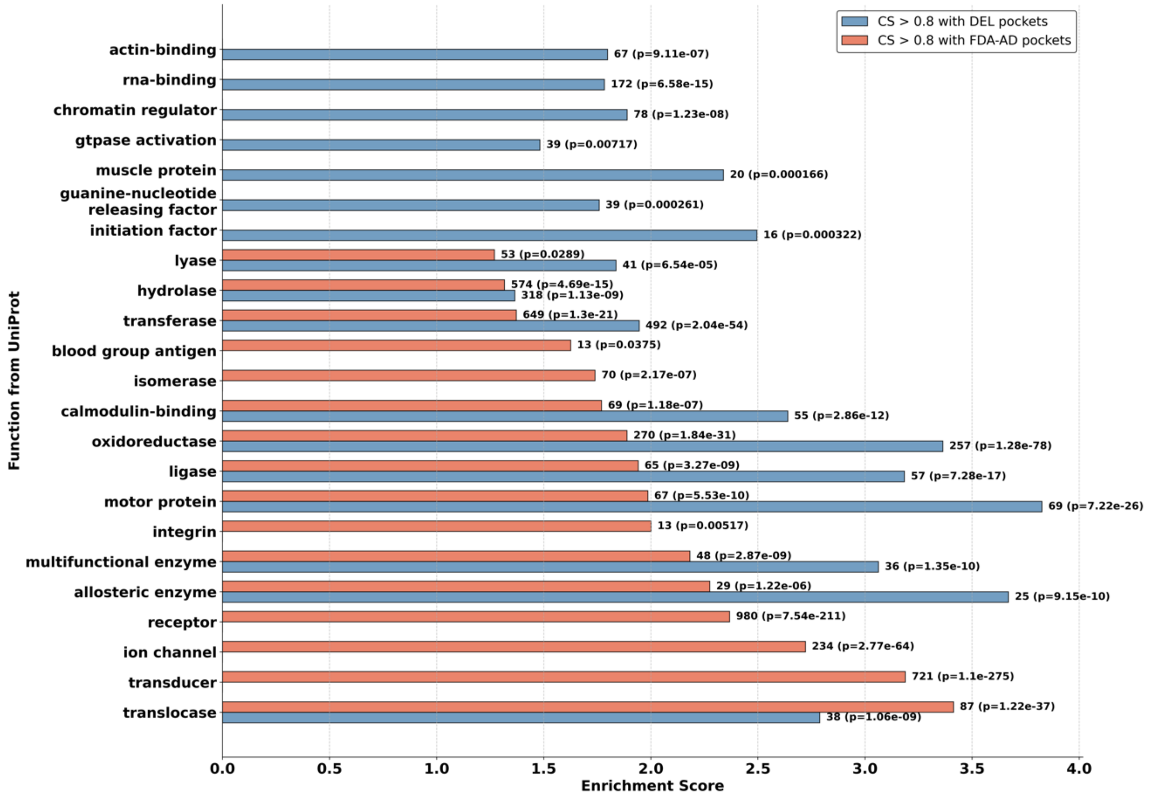

Figure S18. Comparative analysis of human protein enrichment using pocket similarity with either DEL or FDA-AD pockets using ErePOC representation (cosine similarity > 0.8). Enrichment scores were calculated as the ratio of the proportion of proteins with a specific functional annotation in the target set relative to the proportion in the background set (all 23,391 human proteins). The target sets comprise 2,739 and 5,124 unique proteins with cosine similarity > 0.8 to DEL and FDA-AD pockets, respectively. Statistical significance was assessed using a one-sided hypergeometric test for over-representation. Bars represent enrichment scores; numerical labels indicate protein counts for each functional category, with exact P values shown in parentheses (uncorrected for multiple comparisons).

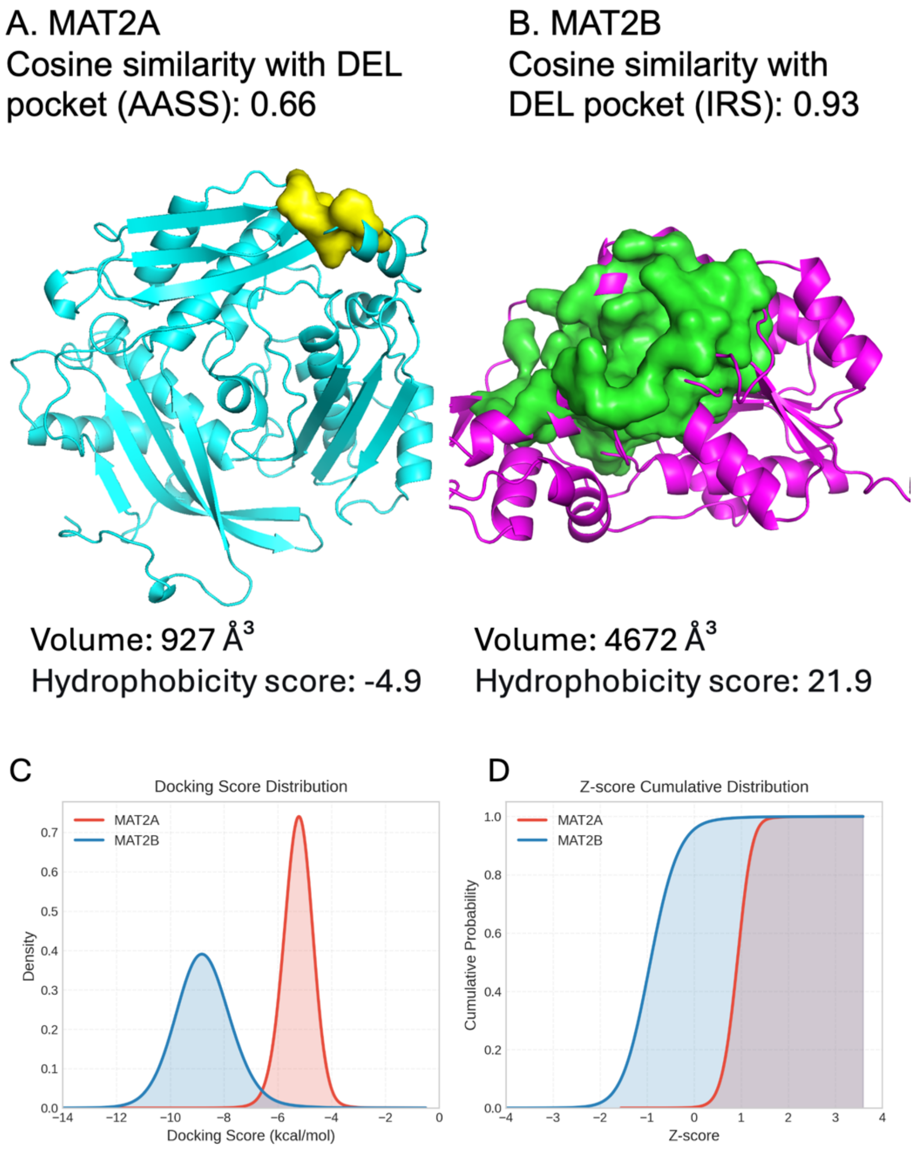

Figure S19. Comparative Analysis of Pocket Properties between MAT2A and MAT2B. A. The structure and pocket of MAT2A; **B.** The structure and pocket of MAT2B; **C.** Distribution of docking scores; **D.** Distribution of docking Z-scores for MAT2A (blue) and MAT2B (red), each docked against a library of 2.8 million small molecules.

Table S1. Summary of datasets included in the study

Dataset	Size
DEL	128
FDA-AD	340
BioLiP2	326,416
Alphafill	293,019
Homo sapiens species	23,391

BioLiP2 dataset: Multiple structures bound to the same ligand are retained intentionally, allowing ErePOC to learn from diverse conformational states of the same ligand across different proteins and crystallization conditions. This enhances functional relevance and improves generalizability.

AlphaFill dataset: Similarly, AlphaFill contains redundant ligand–protein complexes. Redundancy was maintained to expose the model to a broad spectrum of protein contexts and ligand environments. After filtering for steric clashes, 293,019 high-confidence complexes were retained.

FDA-AD dataset: Unique crystal structures were defined as unique FDA drug–protein pairs. Redundant structures for the same drug–target pair were removed, but multiple targets of the same drug are retained.

DEL dataset: A one-to-one mapping was applied by retaining only the highest-affinity DEL hit per target, resulting in 128 unique DEL–target structures.

Table S2. Detailed information of the DEL target protein structures dataset

Index	Target	PDB ID	SMILE for DEL compound
1	3CLpro	7vu6A	Brc1cc([N+](=O)[O-])c(NC2C(NC(=O)c3c4c(cnc3)cccc4)CCCC2)c(C(=O)NC)c1
2	53BP1 TTD	4x34A	O=C(N)C(NC(=O)C=1C(c2c(CNC(=O)c3nccc(N4CC[NH+](C)CC4)c3)cccc2)CC=CC=1)CCCC[NH+]1CCCC1
3	AASS	5l78A	Clc1cnc(OC2CC(NC(=O)C(NC(=O)c3c(SCCS(=O)(=O)c4ccccc4)nccc3)CCC(=O)NC(C(=O)O)CC(=O)N)CCC2)cc1
4	ADAMTS-4	4wk7A	O=C(N(CC)c1cc(C)ccc1)c1ccc(CNc2nc(NC[NH+]3CC[NH+](C)CC3)nc(N3Cc4c(cc(OC)c(OC)c4)CC3)n2)cc1
5	AGP	3kq0A	Clc1c(C2NC(=O)C(CCN=[N+]=N)NC(=O)C(C)Cc3c(C(=O)NCCNC(=O)C2)cccc3)cccc1
6	AGP; CA IX	6rzxA	S(=O)(=O)(N(CC(=O)NCCOCCC(=O)NCCOCCC(=O)NC(C(=O)[O-])CCCCNC(=O)C=Cc1oc(-c2c(C(F)(F)F)cccc2)cc1)c1c(-c2ccccc2)cccc1)c1ccc(C)cc1
7	AT1R	4zudA	Fc1ccc(CC(NC(=O)C(NC(=O)C(NC(=O)C=Cc2cc(O)c(O)cc2)C)CC2CCCCC2)C(=O)OCC)cc1

8	ATAD2	6hi4A	Clc1c(C(=O)NC(C[NH2+]C2CCC([NH3+])CC2)Cc2ccc(C#N)cc2)cc(c(OC)c1)-c1oc(C[NH2+]C(C)c2ccc(C)cc2)cc1
9	ATX	4zgaA	S(=O)(=O)(C)c1ccc(NC2(C)CCN(C(=O)C(NC(=O)c3c(F)ccc(CC)c3)C(C)C)CC2)cc1
10	Aurora A	2xngA	O=C(O)Cn1nnc(C[NH+]2Cc3c(ccc(-c4ccc(C=O)cc4)c3)C2)c1
11	autotaxin	6w35A	FC(F)(F)c1cc(C(=O)NC(C(=O)N2CCC3(N(c4cc5c([nH]nc5)cc4)C(=O)N(C)C3=O)CC2)C(C)C)c(F)cc1
12	β2AR	5x7dA	Fc1ccc(CC(NC(=O)C(NC(=O)C2(NC(=O)C=Cc3cc(O)c(O)cc3)CC2)CC2CCCCC2)C(=O)OCC)cc1
13	β2AR; CysLT	7dhrR	O=C(NCCc1ccc(O)cc1)C1(C)CC2C(C)(CCC3(CC)C4(C)C(C(=O)C=C23)C2(C)C(C)(C(C)(C)C(O)CC2)CC4)CC1
14	β2AR; CysLT	6rz4A	O=C(NCCc1ccc(O)cc1)C1(C)CC2C(C)(CCC3(CC)C4(C)C(C(=O)C=C23)C2(C)C(C)(C(C)(C)C(O)CC2)CC4)CC1
15	BambL	6zfcA	O=C(NC(C(=O)N)Cc1nnn(C2C(O)C(O)C(OC3C(OC4C(O)C(O)C(O)C(C)O4)C(O)C(O)C(CO)O3)C(CO)O2)c1)C(NC(=O)C)Cc1nnn(C2C(O)C(O)C(OC3C(O)C(O)C(O)C(CO)O3)C(CO)O2)c1

16	BCATm	5hneA	Brc1sc(C(=O)N2CC(Oc3c(C(=O)NC)ccc(-c4c(N=S(=O)([O-])C)cccc4)c3)CC2)cc1
17	Bcl-xL	3wixA	Clc1ccc(C(=O)n2c(C)c(CC(=O)NC(CC(=O)[O-])Cc3c4c(ccc3)cccc4)c3c2ccc(OC)c3)cc1
18	BET	7laiA	O(CC(COC)n1c(C2=CN(C)C(=O)C(C)=C2)nc2c1ccc(N1CCOCC1)c2)C
19	β2AR	5a8eA	S(=O)(=O)(N(C(C)C)C)c1cc(C(=O)NC(CC(=O)N)Cc2ccc(C(C)(C)C)cc2)c(Sc2ccc(OC)cc2)cc1
20	BRD2-BD2	5xhkA	O=C(Nc1cc2C3(C(=O)Nc2cc1)C(C(=O)OCCCC)CC1[NH+]3CSC1)C=Cc1cc(OC)c(OC)c(OC)c1
21	BRD4	5t35A	O=C(NC)C(NC(=O)c1c(OC(C)C)cc(C2=CN(C)C(=O)C(C)=C2)cc1)CCCCCNC(=O)COc1c2C(=O)N(C(=O)c2ccc1)C1C(=O)NC(=O)CC1
22	BRD4	5t35D	O=C(NC(C(C)(C)C)C(=O)N1C(C(=O)NCc2ccc(-c3c(C)ncs3)cc2)CC(O)C1)CCC1CCN(c2nc(N(CCC(=O)NC3CC3)C)nc(N(Cc3c(C)n(C)nc3C)C)n2)CC1
23	BRDT-BD1	7uboA	O=C(NC)C(NC(=O)c1ncc(-c2ccccc2)cc1)CC1CCN(C(=O)Cc2cc3C(C)=CC(=O)Nc3cc2)CC1
24	BRDT-BD1, BRDT-BD2	7l9aA	O=C(NC)c1ccc(C(=O)Nc2c(C)ccc(NC(=O)c3cc4c(-c5c(C)cc(N)cc5)nn(C)c4cc3)c2)cc1

25	BTK	4y93A	O=C(C=C)N1C2CN(c3nc(NC)nc(Nc4n[nH]cc4)n3)C(C1)C2
26	c-MET	8ansA	FC(F)c1c(C(=O)N(C)C2c3c(C)ccc(-c4cc5[nH]nc(C)c5cc4)c3CCC2)nccc1
27	c-Src	3el7A	O=C(N)C(NC(=O)CN(C(=O)CCc1ccc(O)cc1)CC1CC1)Cc1cnccc1
28	CA-12	4ww8A	S(=O)(=O)(N)c1ccc(C(=O)NCc2nnn(C3C(O)C(O)C(O)C(CO)O3)c2)cc1
29	CBX2	5epkA	Fc1ccc(CC(NC(=O)c2cc3NC(=O)Nc3cc2)C(=O)NC(C(=O)N)Cc2ccc(-c3ccccc3)cc2)cc1
30	CDK9, CDK7	1ua2A	Clc1c(-c2c3n(nc2)CC(C)(C)C3)cc(NC(=O)Cc2nc(C#N)ccc2)nc1
31	CDK9, CDK7	3blrA	Clc1c(-c2c3n(nc2)CC(C)(C)C3)cc(NC(=O)Cc2nc(C#N)ccc2)nc1
32	CREBBP bromodomain	4tqnA	S(C(=O)NCCNC(=O)c1c2NC(C)CC(=O)Nc2ccc1)C(S)c1ccccc1
33	CREBBP bromodomain	4yk0A	S(C(S)c1ccccc1)CC(=O)NCCNC(=O)c1c2NC(C)CC(=O)Nc2ccc1
34	cyclophilin D (CypD)	7thdA	O=C([O-])C(C(=O)[O-])Cc1ccc(-c2ccc(CC3C(=O)NCc4c(cccc4)CC(=O)NC(C(=O)NCCOCC[NH3+])CCNC(=O)C=CC(=O)N4CC(Cc5ccccc5)(C(=O)N3)CCC4)cc2)cc1

35	DDP-4	2bucA	FC(F)(F)c1n2c(nn1)C1N(C(=O)CC([NH3+])Cc3c(F)cc(F)c(F)c3)C(C2)CC1
36	DDR1	4ckrA	Brc1c2C(C(=O)N(CC(=O)NCC(F)(F)F)c2ccc1)C1CCN(C(=O)c2nc3c([nH]nc3)cc2)CC1
37	MRS; IRS; MetAP; UppS; DHFR	3nz6X	O=C(Nc1nc(C)ccc1)Cc1c(C(=O)[O-])[nH]c2c1cccc2
38	folate receptor (FR) on live regular HeLa cell, EGFR on live A431 cells	5u8lA	S(CCC(NC(=O)C(=NOC)c1nc(N)sn1)C(=O)NC(C(=O)O)Cc1c2c(sc1)cccc2)C
39	sEH, c-KIT, ERα	7te7A	O(C(c1c(C)cccc1)c1c(C)cccc1)C(c1c(C)cccc1)c1c(C)cccc1
40	Fibroblast activation protein (FAP)	1z68A	FC1(F)CN(C(=O)CNC(=O)c2c3c(c(NC(=O)CCC(=O)NC(C(=O)NC(C(=O)NCCNC(=O)CCC(C(=O)O)[NH+]4CC[NH+](CC(=O)O)CC[NH+](CC(=O)O)CC[NH+](CC(=O)O)CC4)Cc4ccccc4)CCCNC(N(C)C)=N)ccc3)ncc2)C(C#N)C1
41	FKBP	5d75A	O=C(O)COc1ccc(CC2C(=O)N3C(C(=O)N(C)C(CC(C)C)C(=O)N(C)CCC=CC(=O)OCC(C)(C)C(=O)C(=O)N4C(C(=O)OC(CCc5cc(OC)c(OC)cc5)c5cc(NC(=O)CCC(=O)NC(c6ccccc6)C(=O)N2)ccc5)CCCC4)CCC3)cc1

42	folate receptor (FR) on live regular HeLa cell, EGFR on live A431 cells	4kmzA	F[F+2]1C=C2C(=O)C(C(=O)NC(C(=O)NC(C(=O)O)(C)c3c4c(ccc3)cccc4)Cc3c4c(sc3)cccc4)=C[NH+]3C(C)CCC(=C23)C1
43	Furin	7lcuA	Clc1cc(Cl)cc(-c2nc(Oc3cnc(N4CC[NH+](C)CC4)nc3)cc(C[NH+]3CCC(CC(=O)[O-])CC3)c2)c1
44	GID4	7u3jA	O=C(NC)C(NC(=O)C(NC(=O)C[NH2+]Cc1c(OC)cc(OC)cc1)c1sccc1)CCc1ccccc1
45	GSK-3β	1q5kA	O=C(NCC1CCC(C(=O)NC)CC1)c1c(-c2ccc(OC)cc2)ocn1
46	HAO1	6w4cA	O=C([O-])c1n[nH]c2c1cc(NCc1c(O)c(-c3onc(N(C)C)n3)ccc1)cc2
47	HDAC6	5ef7A	O=C(NO)C=Cc1ccc(C[NH2+]CCc2c(C)[nH]c3c2cc(C(=O)N(CC(=O)NCCc2c4c([nH]c2)cccc4)C)cc3)cc1
48	HSA	1bj5A	O=C(OC1CCN(C(=O)c2ccc(-c3[nH]c4c(n3)cccc4)cc2)CC1)Nc1cc(C(=O)NCCOCCOCC[NH3+])ccc1
49	Hsp70	4po2A	O=C(NC)C(NC(=O)c1ccc(NC(=O)CCC(=O)c2ccc(-c3ccccc3)cc2)cc1)CNC(=O)c1c(O)ccc(NC(=O)c2c(C)onc2)c1
50	hTNF-α	6ooyA	O=C1C(O)=C(c2ccc(O)cc2)Oc2c1c(O)cc(O)c2

51	IDE	4lteA	O=C(c1ccc(CC2C(=O)NCCCCC(C(=O)N)NC(=O)C=CC(=O)NC(CCCC[NH3+])C(=O)NC(CC3CCCCC3)C(=O)N2)cc1)c1ccccc1
52	IDO1	5ek4A	Brc1cc2C(NC(=O)c3c4c(cn(COP(=O)(O)O)c4)ccc3)CC3(Oc2cc1)CCN(C(=O)OCC)CC3
53	IL-2	1m48A	O=C([O-])C(NC(=O)CCn1c(C)cc2c1cccc2)Cc1ccc(C#CCCC)cc1
54	IL-17A	5vb9A	Fc1cc2OCC3(C(C(NC(=O)C4=C(CC)NON4)C(=O)Nc4cnc(-c5c(C)[nH]nc5C)cc4)c2cc1)CC3
55	InhA NADH	5g0sA	O=C(NC)CN1C(=O)C(Cc2ccccc2)N(C(=O)CC2CCC(NC(=O)Cc3c(C)c4c(s3)cccc4)CC2)CC1
56	Insulin Receptor	2z8cA	O=C1[NH+]2C(=Nc3c1cccc3)c1[nH]c3c(c1CC2)cccc3
57	Integrin LFA-1	1rd4A	Brc1ccc(C(Nc2nc(NC3(C(=O)NCC)Cc4c(cccc4)C3)nc(N3C4CN(C(=O)c5cc(C(F)(F)F)ccc5)C(C3)C4)n2)C)cc1
58	MRS; IRS; MetAP; UppS; DHFR	1jzqA	Clc1cc(Cl)cc(N2CCN(C(=O)c3cc(C(C)(C)C)cc(C[NH2+]Cc4cc(C(=O)NC)ccc4)c3)CC2)c1
59	JAK3	5ttuA	ClCC(=O)Nc1cc(CNc2nc(NC)nc(Nc3n[nH]c(C(C)(C)C)c3)n2)ccc1

60	JNK1	4l7fA	Fc1c(F)ccc(C(=O)C=CC(=O)NCCOCCOCCNC(=O)c2c(Oc3ccccc3)nccc2)c1
61	KMO	4j36B	Clc1c(C(=O)N(C)C2C(C(=O)O)C=C(F)C(Cl)=C2)nc(N2Cc3c(ccc(OC)c3)C2)s1
62	KRAS mutant G12C	6n2kA	Clc1c2c(N3Cc4nc(OCC56[NH+](CCC5)CCC6)cc(N5CC(CC#N)N(C(=O)C(F)=C)CC5)c4CC3)cccc2ccc1
63	LC3B	2zjdA	O=C([O-])CC(n1c(-c2cc(O)c(O)c(C=O)c2)[nH+]c2c1ccc(C(=O)NC)c2)c1cc(Oc2ccccc2)ccc1
64	LpxA	5dg3B	Clc1c(SCC(=O)N(CC(=O)NC)Cc2ccc(-n3ncnc3)cc2)cccc1
65	MAP2K6	8p7jA	Clc1c(Cl)c(C(=O)C(=C)CC)ccc1OCC(=O)O
66	Mcl-1; Bcl-2; Bcl-XL	6oqdA	Clc1cc2c(-c3c(OCC(=O)NC4(C(=O)NC(C(=O)N)CNC(=O)C(NC5=NS(=O)(=O)c6c5cccc6)C(C)C)CC4)cccc3)n[nH]c2cc1
67	MDM2, hTEAD4	4j3eA	Clc1cc2[nH]cc(C(N(C(=O)CCC(=O)OC(C)(C)C)Cc3nnn(CC(=O)Nc4c(C)cc(C)cc4)c3)C(=O)NC3CCCCC3)c2cc1
68	MEK2	1s9iA	Fc1c(-c2ccc(C(=O)C(=C)CC(=O)N3C(C(=O)N)CC(Oc4ccccc4)C3)cc2)ccc(F)c1
69	Mer, Axl	7aw2A	Clc1c(-c2ccc(-c3oc(N(CC4=Cn5c(ncc5)C=C4)C)nn3)cc2)cccc1

70	Mer, Axl	7aazA	O=C(NC1C(OCc2ccc(-c3ccc(C[NH+]4CC[NH+](C)CC4)cc3)cc2)CCC1)c1c(N)ncc(-c2cn(C)nc2)c1
71	MRS; IRS; MetAP; UppS; DHFR	3d27A	Clc1c2n(Cc3[nH]c(C)nn3)c(C(Cc3cc4OCOc4cc3)C)nc2ccc1
72	MIF muclesse	6c5fB	Fc1cc2C(CCc3cc(OC)c(OC)cc3)OC(=O)C3N(C(=O)C(=O)C(C)(C)COC(=O)C=CCCN(C)C(=O)C4N(C(=O)C(COC(C)(C)C)N(C)C(=O)C(c5ccccc5)NC(=O)C(C)N(C)C(=O)COc(c1)c2)CCC4)CCCC3
73	MMP-3	1g49B	S(=O)(=O)([O-])c1c(C=Cc2c(S(=O)(=O)[O-])cc(NC(=O)C)cc2)ccc(NC(=S)NCc2ccc(CNC(=O)CCc3ccc(OCC4CCCCC4)cc3)cc2)c1
74	Mpro	8skhA	FC(F)(F)C1=CC(C2C(C(=O)NC(C(=O)NC)Cc3ccccc3)CN(C(=O)C(NC(=O)C=C)CC(=C)C)C2)CC=C1
75	Mps1	5z33A	FC(F)(F)Oc1cc(c(OC)cc1)-c1c2nc(C(=O)NC(C(=O)NC)Cc3c4c([nH]c3)cccc4)ccc2cnc1
76	MRS; IRS; MetAP; UppS; DHFR	7wpiA	Brc1c(O)cc(-c2n(C(CNC(=O)COc3c(C(C)(C)C)cccc3)c3cc(OC)c(OC)cc3)c3c(n2)cc(C(=O)NC)cc3)cc1
77	Naa50	7ojuA	O=C(NC)C(NC(=O)C1NC(=O)CCC1)CC1CCN(C(=O)c2n(C)nc(C(C)(C)C)c2)CC1

78	NK3	7ukzA	O=C(NC)c1cc2nc(n(CC)c2cc1)-c1c(-c2c(C)cc(C)cc2)cccc1
79	NMP-2	1qibA	O=C(NCCNC(=O)CCc1c(C)n(C2=NC(C)=CC(=O)N2)nc1C)CC(=O)C=CCCCCNC(=O)c1cc(O)cnc1
80	OGT	4n39A	O=C(NC)CC(NC(=O)c1cc(-c2ccc(-c3occc3)cc2)cnc1)c1ccc(-c2ccccc2)cc1
81	OXA-48 Carbapenemase	6pt5A	S(=O)(=O)(O)C1CCN(c2nc(N3CCN(c4c(OCC)cccc4)CC3)ncn2)CC1
82	p38 MAP	1cm8A	O=C(N)C(NC(=O)c1ccc(CNC(=O)c2c(N)n(-c3ccccc3)nc2)cc1)CCC1CCCCC1
83	p38α MAP	7pvuB	O=C(NCc1ccc(C(=O)NCCCC2CCCCC2)cc1)c1c(N)n(-c2ccccc2)nc1
84	p38α MAP	5larA	O=C(N)C(NC(=O)c1ccc(CNC(=O)c2c(N)n(-c3ccccc3)nc2)cc1)CCC1CCCCC1
85	p300/CBP	7ss8A	Clc1ccc(C2(C(=O)N3C(C(=O)NCc4cc(C(=O)NC)ccc4)CCC3)CCCC2)cc1
86	PAD4	1wd8A	O=C(N1CC([NH3+])CCC1)c1cc2nc(n(C)c2cc1)-c1n(C)c2ncccc2c1
87	PAK4, 2-epimerase	8ahgA	O=C(NC)c1c2c(C(C)C)n[nH]c2cnc1
88	PAK4, 2-epimerase	8aheA	S(=O)(=O)(NC)c1c(C)n(-c2ccccc2)nc1

89	PAR2	5nddA	O=C(NC1c2c(C(C(=O)NC)CC1)cccc2)C(NC(=O)c1nnn(Cc2ccccc2)c1)CC1CCCCC1
90	PARP1	7kkpA	O=C1c2c(O)cc(O)cc2OC(c2cc(O)c(O)cc2)=C1
91	PARP10	5lx6A	O=C(NC)C(NC(=O)c1cc2c(C(=O)CCC2)cc1)CNC(=O)CCC=1C(=O)Nc2c(N=1)cccc2
92	PARP1; PARP15; SIRT6	7pw3A	O=C(N)C(NC(=O)c1cc(C)ccc1)CNC(=O)c1cc(C(=O)N)c(C(=O)N)cc1
93	PCAF	3uvwA	Clc1c(Cl)c(C(=O)C(=C)CC)ccc1OCC(=O)NC(C(=O)N)COCCCCCCCCCC
94	PDE12	4z0vA	O=C(N1C(CO)C(c2ccccc2)OCC1)c1cc2[n+](C)c(-c3c4c([nH]c3)cc(C#N)cc4)[nH]c2cc1
95	PI3K	3apcA	O=C(O)C(NC(=O)CCC(NC(=O)C1=C(C)n2nc(C)cc2N=C1)C(=O)Nc1cc(OC)c(OC)c(OC)c1)CCC(=O)N
96	PI3K (WT/H1047R)	4yknA	S(=O)(=O)(N=C1SC(C)=NN1)c1ccc(NCc2cc(C(=O)[O-])cc(-c3cnc(OC)cc3)c2)cc1
97	Pin1	2xpbA	ClCC(=O)N1CCN(c2nc(NC(c3cc(S(=O)(=O)C)ccc3)c3ccccc3)nc(NC)n2)CC1
98	PqsE Thioesterase	7kgwA	Clc1cc(OC2CN(C(OC(C)(C)C)=O)CCCC2)cc(NC(=O)c2c(C(=O)O)cccc2)c1
99	prolyl hydroxylases	5c5tA	O=C([O-])c1ncn(C2=NC(=O)NC(NCc3ccc(-c4ccccc4)cc3)=N2)c1

100	prion protein (PrP)	4ma7A	O=C(NCCOCCOCC=N)C1NC(=O)C2CN(C(=O)CCCN(C)C(=O)C3N(C(=O)C=CC(=O)NCCC1)CCC3)CC2
101	PSMA	1z8lA	O=[N+]([O-])c1c(N2CCCC2)ccc(C(=O)NCC(NC(=O)CC23CC4CC(C2)CC(C3)C4)C(=O)NC)c1
102	RIP2	5j79A	Clc1c(O)cc(NCc2cc(C(=O)NCCOC)cc(-c3cc4[nH+]ccc(Nc5cc(O)c(Cl)cc5)c4cc3)c2)cc1
103	ROCK2	6ed6A	O=C(N1C(c2ccccc2)C[NH2+]CCC1)c1cc2c(cncc2)cc1
104	RORγ Reporter (Gal4)	6q2wA	FC(F)(F)C(O)(C(F)(F)F)c1ccc(CN(c2c(F)c(NCC3C(O)C[NH+](CC(=O)N)CC3)ncn2)C2CC2)cc1
105	RSA; BSA; HSA	6wuwA	FC(F)(F)c1cc(CC(=O)NC(C(=O)NC)CNC(=O)Cc2cc([N+](=O)[O-])ccc2)ccc1
106	RSV N protein	4uccA	Brc1sc(CC2NC(=O)C(Cc3ccccc3)NC(=O)C3N(C(=O)Cn4nnc(c4)CC(C(=O)N)NC(=O)C(C(CC)C)CNC(=O)C(Cc4cc(OC)ccc4)NC(=O)c4cc(NC2)ccc4)CCC3)cc1
107	sEH	1zd4A	FC(F)(F)c1c(CNC(=O)C2CC(Nc3nc(NC)nc(N4CC[NH+](C)CC4)n3)CCC2)cccc1
108	sEH, c-KIT, ERα	5mwaA	Clc1c(Sc2ccc(NC(=O)C3CCN(C(=O)c4ccc(F)cc4)CC3)cc2)cccc1

109	Sirt3	4fvtA	O=[N+]([O-])c1c(C(O)C)cc(OC)c(OCCCC(=O)NC2c3c(cc(OCC(=O)NCCCC)cc3)CCc3c2cccc3)c1
110	SIRT3	4jsrA	O=C(N(CCCN1c2c(cccc2)CCc2c1cccc2)C)CCC(C)(c1ccc(O)cc1)c1ccc(O)cc1
111	SIRT5	6ljmA	O=C(OC(C)(C)C)NC(CC(=O)NCCCCCC(=O)N(CCCN1c2c(cccc2)CCc2c1cccc2)C)Cc1ccccc1
112	SOS1	5oviA	O=C(OC(C)(C)C)N(Cc1c(-c2cc(C(NC3=NNC(=O)c4c3cc(N3CC[NH+](C)CC3)nc4)C)sc2)cccc1)C
113	Src kinase	2srcA	Fc1ccc(CC2C(=O)NC(Cc3ccc([N+](=O)[O-])cc3)C(=O)NCCCC(C(=O)[O-])NC(=O)C=CC(=O)NC(CC3CCCCC3)C(=O)N2)cc1
114	STING	8gt6A	S(C)c1c2n(CC=CCn3c(NC(=O)c4c(CC)nc(C)s4)nc4c3c(OCCC[NH+]3CC[NH+](C5CC5)CC3)cc(C(=O)N)c4)c(NC(=O)c3n(CC)nc(C)c3)nc2cc(C(=O)N)c1
115	Streptavidin	1lcvA	O=C(N(Cc1ccc(C(=O)N(Cc2sc(C[NH+](Cc3ccc(C(=O)N)cc3)C)cc2)C)cc1)CC1CCOCC1)c1ccc(C[NH2+]CC2CCOCC2)cc1
116	TAK1	4l53A	FC(F)Oc1c(CN(C(=O)c2cc(C(=O)N3CCCC3)[nH]c2)CC(=O)NC)cccc1

117	Tankyrase 1	6qxuA	O=C(NCCc1onc(-c2ncccc2)n1)c1c(N2C(=O)NC(=O)CC2)cccc1
118	YAP-Binding domain of TEAD1	8cuhA	Clc1c(C[NH2+]C(C(=O)NC(C(=O)N)c2ccccc2)C2Cc3c(cccc3)C2)c(O)cc(Cl)c1
119	Thrombin	1dwbH	O=C(N(C)C1c2c(OCC1)cccc2)NC(C(=O)N1CC(C(=O)NC)C(c2ccc(OC)cc2)C1)Cc1c(CNC(=[NH2+])N)cccc1
120	TNKS1; HSA	3uh2A	FC1(F)Oc2c(O1)ccc(C(=O)NCc1cc(C(=O)N)cc(CNC(=O)CCc3cc(OC)c(OC)c(OC)c3)c1)c2
121	tankyrase 1 (TNKS1)	4i9iA	O=C(NC)C(NC(=O)c1cnc2c(c1)cccc2)CNC(=O)CCN1C(=O)NC(=O)c2c1cccc2
122	TRK	5wr7A	Fc1cnc2c(c1)C1N(C3=NC=4[NH+](N=CC=4C(=O)NC(C)CC2)C=C3)CCC1
123	TRKA	6iqnA	Fc1cc2c(OCCC(C)NC=3C(C#N)=CNC=4C=3NC(N3C2CCC3)=CC=4)cc1
124	Trypsin	2staA	S(=O)(=O)(NC(C(=O)NC(C(=O)NCc1ccc(C(=[NH2+])N)cc1)C)CNC(=O)Cc1cc2c(cc1)cccc2)c1ccccc1
125	MRS; IRS; MetAP; UppS; DHFR	5kh5B	Clc1c(Cl)cc(C(=O)NCc2ccc(C(C)(C)C)cc2)c(C(=O)NC(C(=O)NC)C(C)(C)C)c1
126	VCP/p97	4kdiA	SC1=[NH+]c2c(C(=O)N1)ccc(C(=O)Nc1ccc(C=CC(=O)N)cc1)c2
127	Wip1	1a6qA	Clc1cc(NCc2sc(C(=O)NC(C(=O)NCCOC)CC3CCCCC3)cc2)ccc1
128	Z α1-antitrypsin	2qugA	O=C(NC(C(O)c1ccccc1)CCC)c1cc2[nH]ccc2cc1
