# Supplementary material for: Deciphering DEL pocket patterns through contrastive learning
Source: Nat Commun. 2026 Feb 16;17:2810. doi: 10.1038/s41467-026-69663-y (PMC13022000; doi:10.1038/s41467-026-69663-y)
Supplement: Supplementary file 2 — Reporting Summary [file 41467_2026_69663_MOESM2_ESM.pdf]

nature portfolio

Corresponding author(s): Jing Huang, Westlake University, huangjing@westlake.edu.cn

Last updated by author(s): Dec 25, 2025

Reporting Summary

Nature Portfolio wishes to improve the reproducibility of the work that we publish. This form provides structure for consistency and transparency in reporting. For further information on Nature Portfolio policies, see our Editorial Policies and the Editorial Policy Checklist.

Statistics

For all statistical analyses, confirm that the following items are present in the figure legend, table legend, main text, or Methods section.

n/a	Confirmed	
☐	☒	The exact sample size (*n*) for each experimental group/condition, given as a discrete number and unit of measurement
☐	☒	A statement on whether measurements were taken from distinct samples or whether the same sample was measured repeatedly
☐	☒	The statistical test(s) used AND whether they are one- or two-sided<br>*Only common tests should be described solely by name; describe more complex techniques in the Methods section.*
☐	☒	A description of all covariates tested
☐	☒	A description of any assumptions or corrections, such as tests of normality and adjustment for multiple comparisons
☐	☒	A full description of the statistical parameters including central tendency (e.g. means) or other basic estimates (e.g. regression coefficient) AND variation (e.g. standard deviation) or associated estimates of uncertainty (e.g. confidence intervals)
☐	☒	For null hypothesis testing, the test statistic (e.g. *F*, *t*, *r*) with confidence intervals, effect sizes, degrees of freedom and *P* value noted<br>*Give P values as exact values whenever suitable.*
☐	☒	For Bayesian analysis, information on the choice of priors and Markov chain Monte Carlo settings
☐	☒	For hierarchical and complex designs, identification of the appropriate level for tests and full reporting of outcomes
☐	☒	Estimates of effect sizes (e.g. Cohen's *d*, Pearson's *r*), indicating how they were calculated

Our web collection on statistics for biologists contains articles on many of the points above.

Software and code

Policy information about availability of computer code

Data collection

BioLiP2 database (https://zhanggroup.org/BioLiP/)
AlphaFill database (https://alphafill.eu/)
AlphaFold database (https://alphafold.ebi.ac.uk/download)
Zenodo (DOI: 10.5281/zenodo.18033921) for processed datasets and target protein lists
DELOpen platform (https://www.delopen.org/), for DEL dataset analyses
NIMBioS amino acid ratios (https://legacy.nimbios.org//~gross/bioed/webmodules/aminoacid.htm), used to analyze enrichment of amino acids in benchmark datasets
PDB (Protein Data Bank) (https://www.rcsb.org/
DrugBank (https://www.drugbank.ca/), for FDA-approved drug information
), for experimentally determined protein structures used in the study

Data analysis	Fpocket 3.0, for pocket characterization.<br>Arpeggio (refactored and expanded version maintained by the PDBe team, version not specified), for protein–ligand interaction analysis.<br>RDKit 2024.03.2, for cheminformatics processing.<br>Open Babel 3.1.1, for molecular format conversions.<br>AutoDock Vina 2.0, for docking calculations.<br>MGLTools 1.5.7, for preparation of docking inputs.<br>Custom code and algorithms (ErePOC v1.0), used for pocket representation learning, lipid-binding protein prediction, and downstream analyses. The code and processed inputs required to reproduce all results reported in this manuscript are publicly available at https://github.com/JingHuangLab/ErePOC.<br>All analyses were performed following the procedures described in the Methods section.

For manuscripts utilizing custom algorithms or software that are central to the research but not yet described in published literature, software must be made available to editors and reviewers. We strongly encourage code deposition in a community repository (e.g. GitHub). See the Nature Portfolio guidelines for submitting code & software for further information.

Data

Policy information about availability of data

All manuscripts must include a data availability statement. This statement should provide the following information, where applicable:

- Accession codes, unique identifiers, or web links for publicly available datasets
- A description of any restrictions on data availability
- For clinical datasets or third party data, please ensure that the statement adheres to our policy

The datasets generated and analyzed in this study have been deposited in Zenodo under DOI 10.5281/zenodo.18033921, including processed datasets, raw data supporting quantitative figures, and PyMOL session files for structural visualization. BioLiP2, AlphaFill, and AF2-predicted protein structure data are publicly available from the BioLiP, AlphaFill, and AlphaFold databases at https://zhanggroup.org/BioLiP/
, https://alphafill.eu/
, and https://alphafold.ebi.ac.uk/download
, respectively. All custom source code and algorithms, together with the processed data required to reproduce the results, are publicly available at https://github.com/JingHuangLab/ErePOC
. There are no restrictions on access.

Research involving human participants, their data, or biological material

Policy information about studies with human participants or human data. See also policy information about sex, gender (identity/presentation), and sexual orientation and race, ethnicity and racism.

Reporting on sex and gender	Not applicable
Reporting on race, ethnicity, or other socially relevant groupings	Not applicable
Population characteristics	Not applicable
Recruitment	Not applicable
Ethics oversight	Not applicable

Note that full information on the approval of the study protocol must also be provided in the manuscript.

Field-specific reporting

Please select the one below that is the best fit for your research. If you are not sure, read the appropriate sections before making your selection.

☒ Life sciences ☐ Behavioural & social sciences ☐ Ecological, evolutionary & environmental sciences

For a reference copy of the document with all sections, see nature.com/documents/nr-reporting-summary-flat.pdf

Life sciences study design

All studies must disclose on these points even when the disclosure is negative.

Sample size	Not applicable
Data exclusions	Not applicable
Replication	Not applicable

Randomization	Not applicable
Blinding	Not applicable

Reporting for specific materials, systems and methods

We require information from authors about some types of materials, experimental systems and methods used in many studies. Here, indicate whether each material, system or method listed is relevant to your study. If you are not sure if a list item applies to your research, read the appropriate section before selecting a response.

Materials & experimental systems

n/a	Involved in the study
☒	☐ Antibodies
☒	☐ Eukaryotic cell lines
☒	☐ Palaeontology and archaeology
☒	☐ Animals and other organisms
☒	☐ Clinical data
☒	☐ Dual use research of concern
☒	☐ Plants

Methods

n/a	Involved in the study
☒	☐ ChIP-seq
☒	☐ Flow cytometry
☒	☐ MRI-based neuroimaging

Plants

Seed stocks	Not applicable
Novel plant genotypes	Not applicable
Authentication	Not applicable
